# Construction Notes

**Published:** 1895-10-12

**Authors:** 


### CONSTRUCTION NOTES.

**Aberdeen City Hospital Extensions.**

Part of the alterations here effected have been to remove the main entrance to Urquhart Road, where it is situated in the middle of the frontage, whence a handsome ornamental iron railing, on a dressed granite basement, runs along the frontage on either side for a distance of nearly 500 ft. At the main extreme stands the new lodge, a neat granite building ornamented with an effective dwarf tower. In this building, besides gatekeeper's accommodation, is a public waiting-room, and there has been fitted up telephonic communication with all the wards as well as with the administrative block. On the right of the main gateway, and running due north and south, are pavilions Nos. 2 and 3, quite separate from each other; and on the left hand, with a considerable expanse of lawn between, another pair of pavilions, Nos. 3 and 4, running parallel with the other two. Directly in front, and thirty yards from the gateway, stands the administrative block with the small-pox pavilion beyond; and far to the left, in the eastmost position of the grounds, are situated the new laundry block, mortuary, &c. Each pavilion is capable of accommodating in all some 70 or 80 patients; the end of each of these is set apart as a male ward, the other end as a female ward, and the space between is devoted to the accommodation of children. In this central portion also are a few rooms set apart for special cases should necessity arise; and the nurses' room, also situated in the central portion of the pavilion, commands, through plate-glass windows, a ready view of every part of the building. The interior arrangements are most thoroughly considered. The pavilions are all one storey high, but the square granite tower that rises from the ends gives an enhanced grace to the appearance of the structure. The administrative block has ample and roomy accommodation. The main door gives access to a roomy vestibule, on the left side of which are the physician's duty-rooms and offices, and on the right-hand side a waiting-room. A transverse passage across the entrance-hall opens on the right to the resident physician's dispensary and laboratory, photographic and dark room, physician's bed-room, sitting-room, and other accommodation. On the left hand the transverse passage leads to the matron's sitting-room, bed-room, and nurses' dining-room. Telephonic communication is fitted up throughout. Passing further along the main corridor into the new portion of the block the kitchen of the hospital is arrived at, a room fully equipped with all the latest appliances. The upper part of the block is mainly devoted to the nurses' sitting-room and bed-room accommodation, and in the basement an elaborate heating and ventilating apparatus, driven by a 7-horse power Tangyes' gas engine. The administrative block is amply provided with store-rooms, lavatory, and bath-room accommodation. The small-pox pavilion is a wooden structure, especially isolated by distance and an intervening wall. At the east end of the grounds, as already indicated, stands the new laundry block, a considerable expanse of buildings of one storey, built in the form of a hollow square. All these departments are furnished with the most modern appliances for the work in hand. In connection with the laundry block there rises a stack some sixty feet high, as in the vicinity is a cinerary for the effective destruction of clothing and other infectious matter. Isolated from the laundry is a mortuary. All these works have cost, owing to their complete and perfect character, a considerable sum—the laundry block alone costing £2,500, and the machinery and fittings £1,000 additionally. The total cost of the improvements, buildings, fittings, &c., is expected to be something like £20,000. The architect for the works is Mr. John Rust, City architect, the mason contractors being Messrs. Leslie Smith and Co.

